# Antidiabetic Drugs for the Risk of Alzheimer Disease in Patients With Type 2 DM Using FAERS

**DOI:** 10.1177/1533317519899546

**Published:** 2020-03-12

**Authors:** Hayato Akimoto, Akio Negishi, Shinji Oshima, Haruna Wakiyama, Mitsuyoshi Okita, Norimitsu Horii, Naoko Inoue, Shigeru Ohshima, Daisuke Kobayashi

**Affiliations:** 1 Faculty of Pharmacy and Pharmaceutical Sciences, Department of Analytical Pharmaceutics and Informatics, Josai University, Sakado, Saitama, Japan; 2 Josai University Pharmacy, Iruma-gun, Saitama, Japan; 3 Faculty of Pharmacy and Pharmaceutical Sciences, Laboratory of Pharmacy Management, Josai University, Sakado, Saitama, Japan

**Keywords:** Alzheimer disease, dementia, type 2 diabetes mellitus, antidiabetics, FDA adverse event reporting system

## Abstract

Alzheimer disease (AD) may develop after the onset of type 2 diabetes mellitus (T2DM), and the risk of AD may depend on the antidiabetic drug administered. We compared the risk of AD among 66 085 patients (≥ 65 years) with T2DM (1250 having concomitant AD) who had been administered antidiabetic drug monotherapy for T2DM who had voluntarily reported themselves in the Food and Drug Administration Adverse Event Reporting System. The risk of AD from the use of different antidiabetic drug monotherapies compared to that of metformin monotherapy was assessed by logistic regression. Rosiglitazone (adjusted reporting odds ratio [aROR] = 0.11; 95% confidence interval [CI]: 0.07-0.17; *P* < .001), exenatide (aROR = 0.22; 95% CI: 0.11-0.37; *P* < .001), liraglutide (aROR = 0.36; 95% CI: 0.19-0.62; *P* < .001), dulaglutide (aROR = 0.39; 95% CI: 0.17-0.77; *P* = .014), and sitagliptin (aROR = 0.75; 95% CI: 0.60-0.93; *P* = .011) were found to have a significantly lower associated risk of AD than that of metformin. Therefore, the administration of glucagon-like peptide 1 receptor agonists and rosiglitazone may reduce the risk of AD in patients with T2DM.

## Introduction

In the United States, Alzheimer disease (AD) currently affects 6.1 million patients in 2017, and this number is expected to rise to 15.0 million patients by 2060.^1^ In addition, extremely high total costs (US$236 billion in 2016) are associated with AD and other forms of dementia.^2^ A potential explanation for the pathogenesis of AD is the amyloid cascade hypothesis. This hypothesis postulates that neurodegeneration in AD is caused by the abnormal accumulation of amyloid β (Aβ)-plaques,^3,4^ which trigger tau protein-induced neuritic injury and neurofibrillary tangles that result in neuronal dysfunction and cell death.^5^ Despite a decrease in Aβ protein levels in patients with AD, clinical drug trials that have been conducted based on this hypothesis have not yet resulted in improvements in cognitive function.^6^ Another hypothesis pertaining to AD pathology is the so-called tau hypothesis.^7^ Under normal conditions, tau acts to stabilize microtubules and control intracellular trafficking.^8,9^ However, tau hyperphosphorylation can contribute to AD by causing neurofibrillary tangles and impeding microtubule assembly.^10,11^

One of the risk factors for AD and dementia is type 2 diabetes mellitus (T2DM),^12,13^ and cross-talk regarding the insulin signaling pathways between AD and T2DM has been reported.^14^ In the brain, insulin is involved in neuronal proliferation, differentiation, and memory formation.^15^ Patients with AD have been reported to display increased insulin resistance in various regions of the brain, such as the cerebellar cortex and hippocampus, regardless of whether they have diabetes.^16,17^ The onset of T2DM is commonly around age 50,^18,19^ while that of AD is generally around age 65 or later.^2^ This suggests that AD may potentially develop after the onset of T2DM. Therefore, the future risk of AD may be altered depending on the antidiabetic drugs used in T2DM therapy.

Metformin has been used as the first line of defense against T2DM, and increasing evidence has proved its efficacy and safety.^20,21^ Metformin has also been reported to lower the risk of dementia.^22^ The aim of the present study was to evaluate potential differences in the risk of AD by comparing various antidiabetic drug monotherapies with metformin monotherapy using the Adverse Event Reporting System (FAERS) that has been made publicly available by the Food and Drug Administration (FDA).

## Methods

### Data Source

The quarterly data files (Q1 2004 to Q3 2018) of the FAERS database published by the FDA (downloaded in December 2018) were used to extract T2DM cases in which patients underwent antidiabetic drug monotherapy. The quarterly data files comprise 7 types of data sets (ie, patient demographic and administrative information, DEMO; drug/biologic information, DRUG; adverse events, REAC; patient outcomes; report sources; drug therapy start and end dates, THER; and indication for use/diagnosis, INDI). The DEMO, DRUG, REAC, and THER files were used in the analyses.

### Antidiabetic Drugs for Analysis

Data from patients who had undergone antidiabetic drug monotherapy for T2DM were subjected to analysis in the present study. The following antidiabetic drugs for T2DM were considered in this analysis: metformin (reference drug), α-glucosidase inhibitors (acarbose and miglitol), sulfonylureas (chlorpropamide, glimepiride, glipizide, glyburide, tolazamide, and tolbutamide), meglinitides (nateglinide and repaglinide), thiazolidinediones (pioglitazone and rosiglitazone), dipeptidyl peptidase-4 (DPP-4 inhibitors; alogliptin, linagliptin, saxagliptin, and sitagliptin), glucagon-like peptide-1 receptor agonists (GLP-1 receptor agonists; albiglutide, dulaglutide, exenatide, liraglutide, and lixisenatide), and sodium-glucose cotransporter 2 inhibitors (canagliflozin, dapagliflozin, and empagliflozin).

### Data Extraction

**Figure 1.**
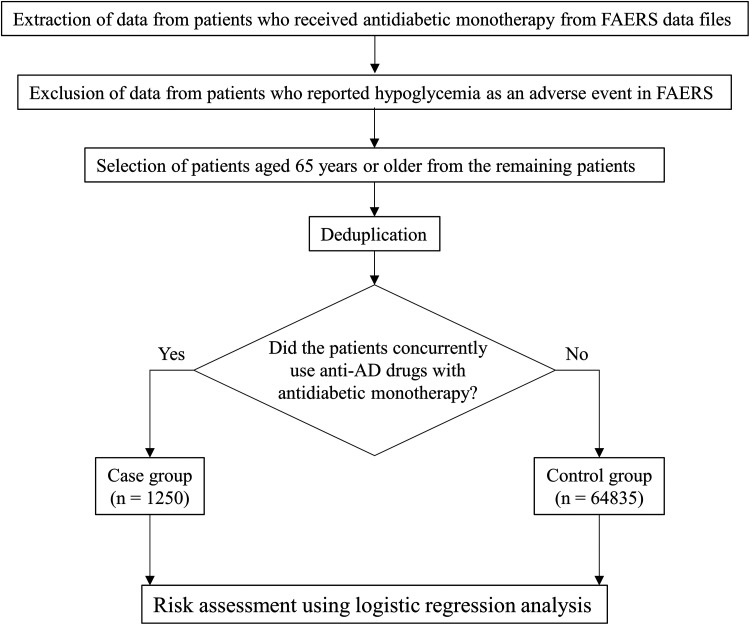
Flowchart of patient data selection for risk assessment. AD indicates Alzheimer disease; FAERS, Food and Drug Administration Adverse Event Reporting System.

Figure 1 presents a flowchart depicting the study procedure, beginning with the selection of patient data reported in FAERS for the risk assessment. The inclusion and exclusion criteria for the analysis cases are given in Table 1. Only data from patients with T2DM who had undergone antidiabetic drug monotherapy were extracted from the FAERS quarterly data files. We only extracted data from patients who were 65 years or older to reflect the common age of the onset of AD.^2^ Given that hypoglycemia is a reported risk factor for dementia,^23,24^ data from patients with hypoglycemia were excluded from the study. Since the analysis involved patients with concomitant AD that developed after the onset of T2DM, we excluded cases in which T2DM developed after the start of a drug used to treat AD. For the purpose of deduplication,^25^ data from patients with a quadruple overlap in age, sex, EVENT_DT (date the adverse event occurred or began), and start date for the different antidiabetic drug monotherapies were excluded. Following deduplication, data from patients with T2DM who had undergone antidiabetic drug monotherapy were divided into 2 groups: patients with concomitant AD (ie, cases) and those without (ie, control). Data from patients with T2DM who had also taken any of the 4 AD therapy drugs (donepezil, galantamine, rivastigmine, or memantine) were assigned to the case group, while those who had not taken any of these drugs were assigned to the control group.

**Table 1.** Inclusion and Exclusion Criteria in Patients with T2DM.

Inclusion	Exclusion
1. Clearly reported age and gender	1. Reported hypoglycemia
2. Age ≥ 65 years	2. On combination therapy of antidiabetic drugs
3. On antidiabetic drug monotherapy	3. Already on AD medication before the start of antidiabetic drug therapy

Abbreviation: AD, Alzheimer disease.

### Covariables to Assess the Risk of AD

Age and gender are not the only potential confounding variables associated with the risk of depression. Common T2DM comorbidities, such as hypertension, dyslipidemia, and cardiovascular disease, can also affect the results of prediction models. These covariables were adjusted in the depression risk model in this study. However, comorbidity status was not available from the data files due to the limited medical histories present in the FAERS INDI files. Thus, the usage of an FDA-approved drug for one of these common comorbidities was considered to be equivalent to having the condition itself. Supplemental Table 1 contains a list of the drugs and the indications for each comorbidity.

### Statistical Analysis

To compare differences in patient backgrounds between the case group and the control group, a 2-tailed Student *t* test was performed to evaluate age differences (continuous data), and a χ^2^ test was used to assess differences due to sex and common T2DM comorbidities (categorical data). In addition, *P* values were corrected by the false discovery rate to avoid any multiple comparison problems. The AD risk of the different antidiabetic drug monotherapies using metformin monotherapy as the control was assessed by multiple logistic regression analysis with a generalized linear model. The use or nonuse of drugs to treat AD was the dependent variable, and use or nonuse of antidiabetic drugs was the independent variable. Age, sex, and common comorbidities of T2DM were adjusted as confounding factors in the logistic regression analysis. As women are at a higher risk than men to develop AD (ie, due to a sex difference),^26^ logistic regression analysis was carried out after a sex-based stratification of case and control groups to assess AD risk with different antidiabetic drug monotherapies for men and women separately. The level of significance was set to 0.05 for all statistical analysis. All statistical analysis in the present study were conducted with R software (version 3.5.1; R Foundation for Statistical Computing, Vienna, Austria).

## Results

### Risk of AD Using Various Antidiabetic Drug Monotherapies

Table 2 shows the backgrounds of patients included in the analysis and the number of patients who had received the different antidiabetic drug monotherapies. A total of 66 085 patients (65 years or older) received antidiabetic drug monotherapy, of which 1250 patients had concomitant AD (case group), while 64 835 patients did not present AD (control group). The case group had a mean age of 79.26 (± 6.90 SD), and female patients accounted for 52.00% of the study participants. The control group had a mean age of 73.69 (± 6.50), and 49.44% were female patients. With regard to patient background, significant differences in age and common T2DM comorbidities were present between the case group and the control group (*P* < .001, respectively), and the case group tended to have a higher number of female patients than the control group (*P* = .131). In both the case and the control groups, most patients had undergone metformin monotherapy (24 090 patients) followed by rosiglitazone monotherapy (11 522 patients). As no patient included in the analysis had undergone lixisenatide monotherapy, we excluded this drug from further analysis.

**Table 2.** Characteristics of All Patients Included in the Analysis.

		All (n = 66 085)		
Characteristics		Case (n = 1250)	Control (n = 64 835)	FDR-Adjusted *P* Value
Age, mean ± SD, years		79.26 ± 6.90	73.69 ± 6.50	<.001
Female (%)		650 (52.00%)	32055 (49.44%)	.131
Common comorbidities of T2DM		n	n	
Hypertension		667	26851	<.001
Dyslipidemia		547	19086	<.001
Cardiovascular disease		304	11041	<.001
Antidiabetic monotherapy	Class	n	n	
Metformin (reference)		578	23512	–
Acarbose	α-glucosidase inhibitors	4	227	.773
Miglitol	α-glucosidase inhibitors	5	139	.486
Chlorpropamide	Sulfonylureas	0	36	1.000
Glimepiride	Sulfonylureas	142	4508	.023
Glipizide	Sulfonylureas	131	3713	.001
Glyburide	Sulfonylureas	86	2014	< 0.001
Tolazamide	Sulfonylureas	0	4	1.000
Tolbutamide	Sulfonylureas	3	51	.204
Nateglinide	Meglinitides	16	363	.055
Repaglinide	Meglinitides	22	697	.350
Pioglitazone	Thiazolidinediones	55	2655	.350
Rosiglitazone	Thiazolidinediones	24	11498	<.001
Alogliptin	DPP-4 inhibitors	16	435	.184
Linagliptin	DPP-4 inhibitors	17	880	.471
Saxagliptin	DPP-4 inhibitors	17	714	1.000
Sitagliptin	DPP-4 inhibitors	99	4803	.184
Albiglutide	GLP-1 receptor agonists	0	845	<.001
Dulaglutide	GLP-1 receptor agonists	7	954	<.001
Exenatide	GLP-1 receptor agonists	11	3147	<.001
Liraglutide	GLP-1 receptor agonists	12	2071	<.001
Lixisenatide	GLP-1 receptor agonists	0	0	1.000
Canagliflozin	SGLT-2 inhibitors	0	894	<.001
Dapagliflozin	SGLT-2 inhibitors	0	146	.097
Empagliflozin	SGLT-2 inhibitors	5	529	.044

Abbreviations: DPP-4, dipeptidyl peptidase-4; FDR, false discovery rate; GLP-1, glucagon-like peptide-1; SD, standard deviation; SGLT-2, sodium-glucose cotransporter-2; T2DM, type 2 diabetes mellitus.

**Figure 2.**
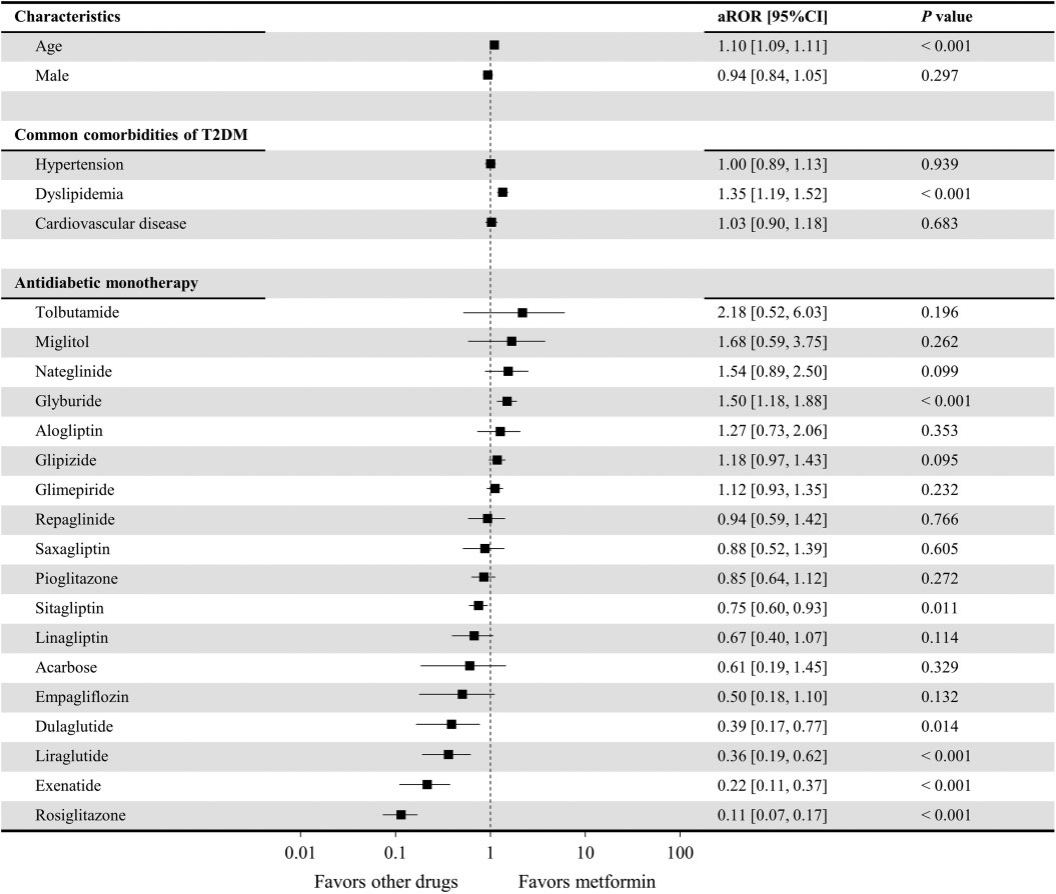
Risk of development of Alzheimer disease using the different antidiabetic drug therapies compared to metformin. Lixisenatide was excluded from the analysis since no relevant cases were extracted (Table 2). Antidiabetic drugs for which there were 0 patients with concomitant Alzheimer disease (chlorpropamide, tolazamide, albiglutide, canagliflozin, and dapagliflozin) were not included in this figure since the adjusted reporting odds ratio (aROR) could not be accurately calculated, and there was a large variance.

Figure 2 shows the risk of AD associated with the different antidiabetic drug monotherapies, including metformin (control). Results from the logistic regression analysis showed no significant relationship between sex and concomitant AD (*P* = .297). However, age (adjusted reporting odds ratio [aROR] = 1.10; 95% confidence interval [CI]: 1.09-1.11; *P* < .001) and dyslipidemia (aROR = 1.35; 95% CI: 1.19-1.52; *P* < .001) were significantly associated with the incidence of concomitant AD. Compared to metformin, glyburide administration resulted in a significantly higher risk of AD (aROR = 1.50; 95% CI: 1.18-1.88; *P* < .001) after adjusting for covariable effects. Nateglinide (aROR = 1.54; 95% CI: 0.89-2.50; *P* = .099) and glipizide (aROR = 1.18; 95% CI: 0.97-1.43; *P* = .095) tended to present a higher risk of AD. In contrast, a significantly lower risk of AD was observed with the administration of rosiglitazone (aROR = 0.11; 95% CI: 0.07-0.17; *P* < .001), exenatide (aROR = 0.22; 95% CI: 0.11-0.37; *P* < .001), liraglutide (aROR = 0.36; 95% CI: 0.19-0.62; *P* < .001), dulaglutide (aROR = 0.39; 95% CI: 0.17-0.77; *P* = .014), and sitagliptin (aROR = 0.75; 95% CI: 0.60-0.93; *P* = .011). As patients who received albiglutide, chlorpropamide, tolazamide, canagliflozin, or dapagliflozin (Table 2) did not have concomitant AD, it was impossible to accurately calculate their aROR values, and thus we do not present these drugs in Figure 2.

### Subgroup Analysis: AD Risk of Antidiabetic Drug Monotherapy by Sex

Of the 66 085 patients (aged 65 or older) who had undergone antidiabetic drug monotherapy, 33 380 were men and 32 705 were women (Table 3). The mean age for men in the case group and control group was 78.69 years (± 6.53; 600 patients) and 73.46 years (± 6.30; 32 780 patients), respectively, and a significant difference in mean age between the 2 groups was present (*P* < .001). The mean age of women in the case group and control group was 79.80 years (± 7.19; 650 patients) and 73.92 years (± 6.69; 32,055 patients), respectively, and a significant difference in mean age was also present between these 2 groups (*P* < .001). In addition, the incidence of T2DM common comorbidities were significantly different between the case and control groups regardless of sex (*P* < .001, respectively). Most female and male patients received metformin monotherapy followed by rosiglitazone monotherapy, which was a common feature between the 2 groups.

**Table 3.** Characteristics of Patients Included in the Analysis, by Sex.

	Male (n = 33 380)			Female (n = 32 705)		
Characteristics	Case (n = 600)	Control (n = 32780)	FDR-Adjusted *P* Value	Case (n = 650)	Control (n = 32055)	FDR-Adjusted *P* Value
Age, mean ± SD, years	78.69 ± 6.53	73.46 ± 6.30	< 0.001	79.80 ± 7.19	73.92 ± 6.69	<.001
Common comorbidities of T2DM	n	n		n	n	
Hypertension	310	13471	<.001	357	13380	<.001
Dyslipidemia	281	10069	<.001	266	9017	<.001
Cardiovascular disease	168	6348	<.001	136	4693	<.001
Antidiabetic monotherapy	n	n		n	n	
Metformin (reference)	265	11 409	-	313	12 103	-
Acarbose	0	105	.313	4	155	1.000
Miglitol	2	86	1.000	3	53	.263
Chlorpropamide	0	6	1.000	0	30	1.000
Glimepiride	65	2429	.482	77	2079	.019
Glipizide	71	1915	.002	60	1798	.150
Glyburide	51	1139	<.001	35	875	.053
Tolazamide	0	3	1.000	0	1	1.000
Tolbutamide	3	31	.079	0	20	1.000
Nateglinide	10	181	.032	6	182	.675
Repaglinide	7	391	.774	15	306	.063
Pioglitazone	28	1500	.482	27	1155	.887
Rosiglitazone	13	6405	<.001	11	5093	<.001
Alogliptin	7	245	.695	9	190	.172
Linagliptin	3	419	.059	14	461	.734
Saxagliptin	7	344	1.000	10	370	1.000
Sitagliptin	51	2338	.927	48	2465	.140
Albiglutide	0	420	<.001	0	425	<.001
Dulaglutide	3	398	.075	4	556	.009
Exenatide	5	1249	<.001	6	1898	<.001
Liraglutide	5	915	<.001	7	1156	<.001
Lixisenatide	0	0	1.000	0	0	1.000
Canagliflozin	0	492	<.001	0	402	<.001
Dapagliflozin	0	68	.593	0	78	.397
Empagliflozin	4	292	.593	1	237	.066

Abbreviations: FDR, false discovery rate; SD, standard deviation; T2DM, type 2 diabetes mellitus.

**Figure 3.**
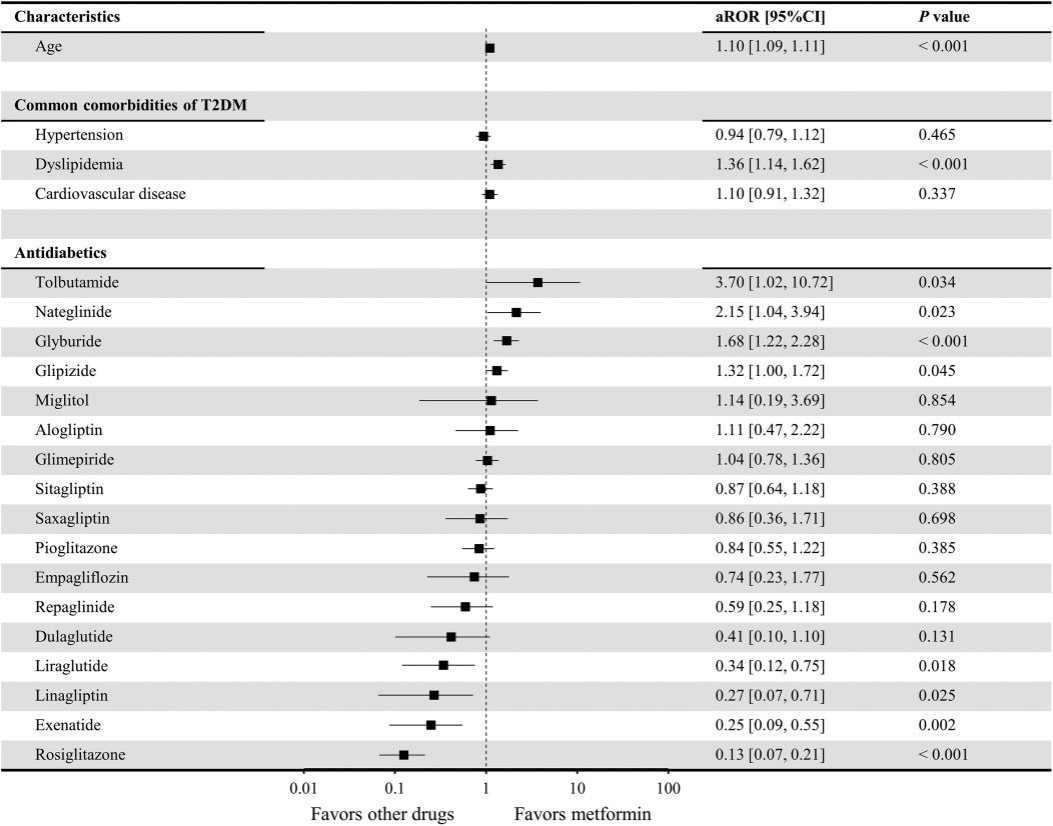
Risk associated with the development of Alzheimer disease with the use of different antidiabetic drug therapies in male patients. Lixisenatide was excluded from the analysis, since no relevant cases were extracted (Table 2). Antidiabetic drugs for which there were 0 patients with concomitant Alzheimer disease (acarbose, chlorpropamide, tolazamide, albiglutide, canagliflozin, and dapagliflozin) are not included in this figure since the adjusted reporting odds ratio (aROR) could not be accurately calculated and large variance was present.

Figure 3 shows the risk of AD when male patients received different antidiabetic drug monotherapies, including metformin monotherapy (control). Age (aROR = 1.10; 95% CI: 1.09-1.11; *P* < .001) and dyslipidemia (aROR = 1.36; 95% CI: 1.14-1.62; *P* < .001) presented significant relationships with the incidence of concomitant AD. Male patients had a significantly higher risk of AD when prescribed tolbutamide (aROR = 3.70; 95% CI: 1.02-10.72; *P* = .034), nateglinide (aROR = 2.15; 95% CI: 1.04-3.94; *P* = .023), glyburide (aROR = 1.68; 95% CI: 1.22-2.28; *P* < .001), and glipizide (aROR = 1.32; 95% CI: 1.00-1.72; *P* = .045) compared to patients in the control group. In contrast, a significantly lower risk of AD was found with rosiglitazone (aROR = 0.13; 95% CI: 0.07-0.21; *P* < .001), exenatide (aROR = 0.25; 95% CI: 0.09-0.55; *P* = .002), linagliptin (aROR = 0.27; 95% CI: 0.07-0.71; *P* = .025), and liraglutide (aROR = 0.34; 95% CI: 0.12-0.75; *P* = .018). No male patients with concomitant AD were identified when acarbose, albiglutide, chlorpropamide, tolazamide, canagliflozin, and dapagliflozin (Table 3) were used, which made it impossible to accurately calculate their aROR values. Therefore, these antidiabetic drugs are not included in Figure 3.

**Figure 4.**
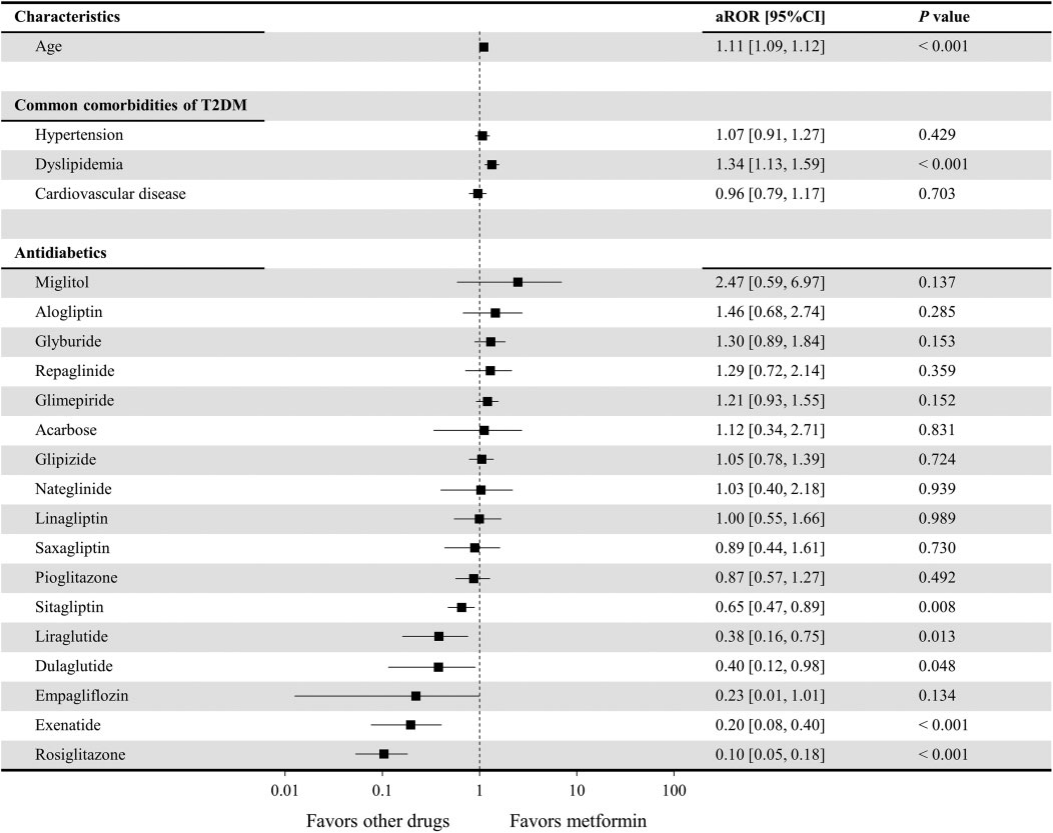
Risk associated with the development of Alzheimer disease with the use of different antidiabetic drug therapies in female patients. Lixisenatide was excluded from the analysis since no relevant cases were extracted (Table 2). Antidiabetic drugs for which there were 0 patients with concomitant Alzheimer disease (chlorpropamide, tolazamide, tolbutamide, albiglutide, canagliflozin, and dapagliflozin) are not included in this figure, as the adjusted reporting odds ratio (aROR) could not be accurately calculated and large variance was present.

Figure 4 shows the risk of AD when female patients received different antidiabetic drug monotherapies, including metformin (control). Age (aROR = 1.11; 95% CI: 1.09-1.12; *P* < .001) and dyslipidemia (aROR = 1.34; 95% CI: 1.13-1.59; *P* < .001) presented significant relationships with the incidence of concomitant AD. Compared to metformin, no antidiabetics were associated with a significantly higher risk of AD in female patients, but a significantly lower risk of AD was associated with rosiglitazone (aROR = 0.10; 95% CI: 0.05-0.18; *P* < .001), exenatide (aROR = 0.20; 95% CI: 0.08-0.40; *P* < .001), liraglutide (aROR = 0.38; 95% CI: 0.16-0.75; *P* = .013), dulaglutide (aROR = 0.40; 95% CI: 0.12-0.98; *P* = .048), and sitagliptin (aROR = 0.65; 95% CI: 0.47-0.89; *P* = 0.008). Since no female patients with concomitant AD were present when albiglutide, chlorpropamide, tolazamide, tolbutamide, canagliflozin, and dapagliflozin (Table 3) were prescribed, it was impossible to accurately calculate their aROR values, and thus they are not included in Figure 4.

## Discussion

In the present study, we investigated the risk of AD with metformin monotherapy and other antidiabetic drug monotherapies. Compared to metformin, a higher risk of AD was associated with glyburide, while a lower risk was associated with GLP-1 receptor agonists and rosiglitazone (Figure 2). All other antidiabetic drugs presented equivalent risks of AD to that of metformin. Metformin, the reference drug in this study, can reportedly cross the blood–brain barrier (BBB).^27^ Metformin is known to reduce levels of Aβ in the brain by lowering the expression of receptor for advanced glycation end products (RAGE), a protein involved in the transport of Aβ across the BBB.^28^ However, metformin is not the only drug known to decrease RAGE expression; glibenclamide and insulin do this as well. As metformin has been reported to reduce the risk of dementia,^22^ the fact that most antidiabetic drugs presented the same risk of AD as metformin suggests that fluctuations in blood sugar levels may be a factor that affects the risk of AD. In fact, it has been reported that hypoglycemia is associated with a higher risk of dementia,^23,24^ and a significant relationship between postprandial hyperglycemia/acute glucose fluctuations and cognitive impairments has been observed.^29,30^ To add, studies of the relationship between the hyperglycemia markers hemoglobin A_1C_ (HbA_1C_) and glycated albumin and the risk of dementia have been published.^24,31,32^ Therefore, the control of blood sugar fluctuations is considered to be very important to lower the risk of AD in patients with T2DM.^33,34^

The results of the present study suggest that GLP-1 receptor agonists present significantly lower risks of AD than metformin. The GLP-1 receptor agonists not only reduce insulin resistance^35,36^ but are also known to be accompanied by a low incidence of hypoglycemia.^20,37^ Liraglutide can pass through the BBB,^38^ and at high doses, exenatide can also pass through the BBB.^39^ Although no studies on the BBB permeability of dulaglutide could be found, dulaglutide was found to provide a more significant reduction in HbA_1C_ than metformin.^40^ The GLP-1 receptor agonists that are transported across the BBB bind to GLP-1 receptors in the brain^41^ and exhibit neuroprotective activity.^42,43^ The GLP-1 receptor agonists may therefore have a lower associated risk of AD than metformin because they combine favorable glycemic control with neuroprotective action in the brain. Given that GLP-1 receptor agonists are injectable formulations, patients require a better understanding of how to use them compared to other antidiabetic drugs.^44^ That is, patients taking GLP-1 receptor agonists may already have higher cognitive function, which might be one of the reasons for the lower risk of AD when patients use GLP-1 receptor agonists.

Rosiglitazone had a significantly lower risk of AD than metformin and the lowest risk of AD of all the drugs included in the analysis. Meanwhile, the risk of AD with pioglitazone was equivalent to that of metformin. Rosiglitazone itself is known to seldom pass through the BBB^45^ but is reported to control Aβ transport to the brain via peroxisome proliferator-activated receptor γ activity.^46^ Similarly, pioglitazone controls Aβ transport to the brain,^47^ and both rosiglitazone and pioglitazone have been reported to display an equivalent reduction of postprandial blood sugar levels and HbA_1C_.^48,49^ This result makes it difficult to use the control of Aβ transport or glycemic control to explain the difference in the associated risk of AD between rosiglitazone and pioglitazone. However, studies have found that rosiglitazone exhibits a therapeutic effect against AD, although this effect has been found to be inconsistent.^50,51^ Rosiglitazone may thus lower the risk of AD, although it is unclear why this drug presented the lowest risk of AD compared to that of the other antidiabetic drugs. In addition, while rosiglitazone has a low risk of AD, thiazolidinediones have been found to increase the risk of heart failure in patients with T2DM,^52,53^ which warrants the use of caution when prescribing rosiglitazone.

Glipizide and glyburide had a significantly higher risk of AD than metformin, while tolbutamide presented the highest risk, although these results were not significant (ROR = 2.11). This suggests that some sulfonylureas may present higher risks of AD than metformin. Multiple reports have shown that the use of sulfonylureas provides less favorable glycemic control than the use of other antidiabetic drugs.^20,38,53-55^ A potential reason for the significantly higher risk of AD associated with sulfonylurea monotherapy is the poor glycemic control that it exhibits.

Male patients with T2DM had a significantly higher risk of AD with nateglinide and sulfonylureas, such as tolbutamide, glyburide, and glipizide than with metformin. Although there have been no reports on a sex-related differences between the risk of AD and these antidiabetic drugs (including metformin), a HbA_1C_-lowering effect of metformin has been reported. Metformin efficacy is affected by sex, but a sex-related difference in the HbA_1C_-lowering effect of sulfonylureas has not been found.^56^ Thus, the high risk of AD in male patients with T2DM treated with sulfonylureas or nateglinide monotherapy may be more due to the relatively greater risk of AD associated with sulfonylurea and nateglinide treatment than due to the better glycemic control of metformin compared to what has been observed in female patients. It has also been suggested that linagliptin and sitagliptin display sex-related differences with regard to the risk of AD. However, as no studies have found a relationship between the risk of AD or glycemic control with these 2 antidiabetic drugs nor sex-related differences, it remains unclear why linagliptin and sitagliptin displayed a sex-related difference with regard to the risk of AD.

### Limitation

A limitation of our study was related to the data available in the FAERS database and was due to patient data in the database being collected via voluntarily reports. Firstly, our results may not only have a reporting bias but may also lack information, such as disease duration or the clinical laboratory values for most patients included in the analysis. The risk of AD in patients with T2DM is known to be affected by the disease duration of T2DM and the baseline values of HbA_1C_.^57,58^ Although we were able to consider age, sex, and common comorbidities of T2DM in the present study, it was impossible to consider disease duration or HbA_1C_ baseline values. Secondly, while GLP-1 analogs were associated with significantly lower AD risk, much fewer patients were taking them than metformin (the reference drug): Accordingly, this finding may have low statistical reliability. Therefore, future studies will need to include these parameters. In addition, given that the present study is a case–control study, our findings need to be verified through cohort studies.

## Conclusion

The objective of this study was to investigate whether the usage of certain antidiabetic drugs was predictive of the future risk of developing AD in patients with T2DM. The use of GLP-1 analogs and rosiglitazone was found to reduce the risk of AD. However, since the FAERS database is comprised only of voluntarily reported adverse events, our findings may be subject to a reporting bias. To address this limitation, we plan to analyze electronic medical records and other real-world data in the future to raise the evidence level of the recommendations of this study.

## Supplemental Material

Supplemental Material, sj-pdf-1-aja-10.1177_1533317519899546 - Antidiabetic Drugs for the Risk of Alzheimer Disease in Patients With Type 2 DM Using FAERS Supplemental Material, sj-pdf-1-aja-10.1177_1533317519899546 for Antidiabetic Drugs for the Risk of Alzheimer Disease in Patients With Type 2 DM Using FAERS by Hayato Akimoto, Akio Negishi, Shinji Oshima, Haruna Wakiyama, Mitsuyoshi Okita, Norimitsu Horii, Naoko Inoue, Shigeru Ohshima and Daisuke Kobayashi in American Journal of Alzheimer's Disease & Other Dementias®
