# Supplementary material for: Antidiabetic Drugs for the Risk of Alzheimer Disease in Patients With Type 2 DM Using FAERS
Source: Am J Alzheimers Dis Other Demen. 2020 Mar 12;35:1533317519899546. doi: 10.1177/1533317519899546 (PMC11005324; doi:10.1177/1533317519899546)
Supplement: Supplemental Material, sj-pdf-1-aja-10.1177_1533317519899546 - Antidiabetic Drugs for the Risk of Alzheimer Disease in Patients With Type 2 DM Using FAERS [file sj-pdf-1-aja-10.1177_1533317519899546.pdf]

Title: Antidiabetic drugs for the risk of Alzheimer's disease in patients with type 2 DM using FAERS

Hayato Akimoto, Akio Negishi, Shinji Oshima, Haruna Wakiyama, Mitsuyoshi Okita, Norimitsu Horii, Naoko Inoue, Shigeru Ohshima, and Daisuke Kobayashi

Supplemental Table 1. List of drugs approved by FDA for common comorbidities of T2DM

No.	Drugs for cardiovascular disease	No.	Antihypertensive drugs	No.	Drugs for dyslipidemia
1	CLOPIDOGREL	1	NIFEDIPINE	1	ATORVASTATIN
2	PLAVIX	2	PROCARDIA	2	LIPITOR
3	PRASUGREL	3	ADALAT	3	CADUET
4	EFFIENT	4	AFEDITAB	4	SIMVASTATIN
5	TICLOPIDINE	5	AMLODIPINE	5	ZOCOR
6	TICLID	6	AMTURNIDE	6	SIMCOR
7	TICAGRELOR	7	AMVAZ	7	VYTORIN
8	BRILINTA	8	AZOR	8	JUVISYNC
9	CILOSTAZOL	9	CADUET	9	FLUVASTATIN
10	PLETAL	10	CONSENSI	10	LESCOL
11	DIPYRIDAMOLE	11	EXFORGE	11	LOVASTATIN
12	AGGRENOX	12	KATERZIA	12	MEVACOR
13	PERSANTINE	13	LOTREL	13	ADVICOR
14	WARFARIN	14	NORVASC	14	ALTOPREV
15	ATHROMBIN	15	PRESTALIA	15	PITAVASTATIN
16	ATHROMBIN-K	16	TEKAMLO	16	LIVALO
17	COUMADIN	17	TRIBENZOR	17	NIKITA
18	JANTOVEN	18	TWYNSTA	18	ZYPITAMAG
19	PANWARFIN	19	NICARDIPINE	19	PRAVASTATIN
20	ABCIXIMAB	20	CARDENE	20	PRAVACHOL
21	REOPRO	21	FELODIPINE	21	ROSUVASTATIN
22	TIROFIBAN	22	LEXXEL	22	CRESTOR
23	AGGRASTAT	23	PLENDIL	23	FENOFIBRATE
24	EPTIFIBATIDE	24	CLEVIDIPINE	24	CLOFIBRATE
25	INTEGRILIN	25	CLEVIPREX	25	ANTARA
26	DABIGATRAN	26	ISRADIPINE	26	ATROMID-S
27	PRADAXA	27	DYNACIRC	27	CLOFIBRATE
28	APIXABAN	28	NIMODIPINE	28	LIPIDIL
29	ELIQUIS	29	NIMOTOP	29	LIPOFEN

30 EDOXABAN
31 SAVAYSA
32 RIVAROXABAN
33 XARELTO
34 FONDAPARINUX
35 ARIXTRA
36 ARGATROBAN

30 NYMALIZE
31 NISOLDIPINE
32 SULAR
33 QUINAPRIL
34 ACCUPRIL
35 ACCURETIC
36 QUINARETIC
37 PERINDOPRIL
38 ACEON
39 PRESTALIA
40 RAMIPRIL
41 ALTACE
42 BENAZEPRIL
43 LOTENSIN
44 LOTREL
45 CAPTOPRIL
46 CAPOTEN
47 CAPOZIDE
48 ENALAPRIL
49 ENALAPRILAT
50 EPANED
51 LEXXEL
52 TECZEM
53 VASERETIC
54 VASOTEC
55 FOSINOPRIL
56 MONOPRIL
57 LISINOPRIL
58 PRINIVIL
59 PRINZIDE
60 QBRELIS
61 ZESTORETIC
62 ZESTRIL
63 TRANDOLAPRIL
64 MAVIK

30 TRICOR
31 TRIGLIDE
32 TRILIPIX
33 EZETIMIBE
34 LIPTRUZET
35 VYTORIN
36 ZETIA

65 TARKA
66 MOEXIPRIL
67 UNIRETIC
68 UNIVASC
69 OLMESARTAN
70 AZOR
71 BENICAR
72 TRIBENZOR
73 VALSARTAN
74 BYVALSON
75 DIOVAN
76 ENTRESTO
77 EXFORGE
78 PREXXARTAN
79 VALTURNA
80 CANDESARTAN
81 ATACAND
82 IRBESARTAN
83 AVALICE
84 AVAPRO
85 LOSARTAN
86 COZAAR
87 HYZAAR
88 AZILSARTAN
89 EDARBI
90 EDARBYCLOR
91 EPROSARTAN
92 TEVETEN
93 TELMISARTAN
94 MICARDIS
95 TWYNSTA
96 FUROSEMIDE
97 LASIX
98 BUMETANIDE
99 BUMEX

100 HYDROCHLOROTHIAZIDE
101 TRIAMTERENE
102 DYAZIDE
103 DYRENIUM
104 MAXZIDE
105 SPIRONOLACTONE
106 ALDACTAZIDE
107 ALDACTONE
108 CAROSPIR
109 EPLERENONE
110 INSPRA
111 PRAZOSIN
112 MINIPRESS
113 DOXAZOSIN
114 CARDURA
